# Purification of Tropomyosin, Paramyosin, Actin, Tubulin, Troponin and Kinases for Chemiproteomics and Its Application to Different Scientific Fields

**DOI:** 10.1371/journal.pone.0022860

**Published:** 2011-08-18

**Authors:** Tomas Erban

**Affiliations:** Laboratory of Proteomics, Department of Stored Product Pest Control and Food Safety, Crop Research Institute, Prague, Czechia; University of Helsinki, Finland

## Abstract

**Background:**

p-aminobenzamidine (p-ABA) is used as a ligand in the purification of many serine proteases and in their removal from heterogeneous samples. Moreover, p-ABA has a potent ability to bind Ca^2+^-binding proteins. The binding ability and use of p-ABA in purification processes is still not fully understood.

**Methodology/Principal Findings:**

A p-Aminobenzamidine (p-ABA) ligand enabled the purification of the panallergenic proteins tropomyosin and paramyosin, as well as actin, tubulin, troponin and several kinases and annexins, with variable specificity depending on the tissue source and slight modifications to the purification process. The high affinity of p-ABA to tropomyosin, paramyosin, actin, troponin and myosin is calcium-dependent, since calcium regulates the function of these proteins. In addition, p-ABA probably simulates phosphorylated serine and therefore purified appropriate kinases. Because p-ABA binds to calcium-dependent proteins, and probably those with binding sites containing serine, it is not a suitable inhibitor of proteolysis during the purification of such proteins. p-ABA is widely used to inhibit proteases during protein purification processes, but it is used in columns here to purify non-protease proteins. Two strategies were applied; the first was the inactivation of proteases that were not of interest using protease inhibitors. The second strategy employed was the use of a Ca^2+^ wash solution to remove calcium-dependent proteins. The removal of calcium-dependent proteins from rabbit hind muscle pointed out even more selective purification. It is possible to obtain two purified samples: a) calcium dependent proteins and b) calcium independent proteins. Moreover, p-ABA may be useful as a model to study processes involving the phosphorylation of serine.

**Conclusion:**

A p-Aminobenzamidine (p-ABA) ligand enabled the purification of non-protease proteins, with variable specificity depending on the tissue source and slight modifications to the purification process. The method is applicable to various scientific branches, but is especially practical for medicinal applications.

## Introduction

Benzamidine and its derivatives are specific competitive inhibitors of trypsin, thrombin, plasmin and all arginine-specific serine proteases. The synthetic inhibitor of serine proteases p-aminobenzamidine (p-ABA) is used as a spectral probe for studying the kinetics of these proteases [1]. In addition, p-ABA has been used for many years as a ligand in the purification of many serine proteases and in their removal from heterogeneous samples [2], [3], [4]. Enterokinase, an enteropeptidase which activates pancreatic trypsinogens, is one of the proteases most often purified using p-ABA [5]. An aspartyl protease has been also purified using the reagent [6].

Tian *et al.* (2006) used p-ABA to characterize proteins that interact with immobilized p-ABA using a chemiproteomic approach and showed that p-ABA has a potent ability to bind Ca^2+^-binding proteins [7]. Therefore, p-ABA, which is commercially available in derivatized column form, may be useful in mass spectrometry-based peptidome research [8].

The objective of this study was to demonstrate novel applications of p-ABA as a ligand in purification processes and for proteomic studies that are applicable to various scientific branches. Using a proteomic approach based on one- and two-dimensional gel electrophoresis together with mass spectrometry analysis were identified purified proteins and their isoforms. Improvements to the purification process have led to improved differentiation of these proteins. The functions of tropomyosins, actins, troponins, 14-3-3 proteins, creatine kinases, annexins and glycogen phosphorylases and invertebrate paramyosin can be studied after the purification/depletion process.

## Materials and Methods

### Reagents

All purification procedures were performed at 4°C on ice, using 0.2-µm-filtered nanopure water (Barnstead, Thermo) throughout. The nondenaturing zwitterionic detergent CHAPS, glycine, iodoacetamide (IAA), dithiothreitol (DTT), agarose (Cat No. A7431) and Bradford reagent were obtained from Sigma-Aldrich (Saint Louis MO, USA). Buffers were prepared from Sigma Ultra purity grade chemicals obtained from Sigma-Aldrich. The HiTrap™ Benzamidine FF column (high sub) (Cat No. 17-5143-01), PD MidiTrap™ G-25 columns (Cat No. 17-5143-01), DeStreak Rehydration solution (Cat No. 18-1168-31), IPG buffer pH 3-10 (Cat No. 17-6000-87) and protease inhibitor mix (Cat No. 80-6501-23) were obtained from GE Healthcare Bio-Sciences AB (Uppsala, Sweden). The buffers used were: (i) Equilibration and wash buffer (0.2 µm filtered phosphate saline buffer 0.01 M, NaCl 0.138 M; KCl - 0.0027 M, pH 7.4); (ii) Binding buffer (equilibration and wash buffer with 1% CHAPS (w/w); (iii) Elution buffer (0.05 M Tris-Glycine, pH 3.0). A 37.5∶1 Acrylamide/bisAcrylamide solution (Cat. No. A3699, Sigma-Aldrich) and tris-glycine-SDS Buffer 10× Concentrate, both from Sigma-Aldrich, were diluted in distilled water for use in SDS-PAGE tris-glycine electrophoresis.

### Biological samples

Eight species of synanthropic acaridid mites and *Blatella germanica* were selected for the study, because of their medical and economical importance. *Oryctolagus cuniculus* was selected for the study to examine usability on pure muscle sample. *Lepidoglyphus destructor*, *Tyroborus lini*, *Dermatophagoides farinae*, *Acarus siro*, *Tyrophagus putrescentiae*, *Blomia tropicalis*, *Glycyphagus domesticus* and *Aleuroglyphus ovatus* were the source of mite proteins. The mites were collected manually using a Camel's-hair pencil. *B. germanica*, reared as previously described (Stejskal, 1997), was the source of *B. germanica* proteins. Cockroaches were collected using forceps and cooled for 5 min in a freezer (−10°C), after which the abdomen was removed with a scalpel. Thigh muscle from the common rabbit, *O. cuniculus* was separated by a dissector and cut into small pieces. *O. cuniculus* was previously used in experiments for production of antibodies (protocol No. 5/09; VÚRV, v.v.i.) and was currently available in the laboratory as cadaver. All samples originated from the Crop Research Institute, Prague.

### Protein extraction

Homogenization of the samples was performed in a sterilized glass Potter-Elvehjem homogenizer (Art. No. 6305; Kartell Labware division, Noviglio, Italy). Briefly, a 0.1-g sample of mite bodies was homogenized using a drilling machine in 1 ml of cold binding buffer with the aid of 20 µL of protease inhibitor mix per 1 mL of mite sample and 10 µL of mix per 0.1 g of *O. cuniculus* or *B. germanica* sample. Each sample was homogenized three times for 2 min each followed by 20 min of cooling on ice. Next, a half volume of extraction buffer was added and homogenization was repeated, three times for 1 min each, and the homogenate was left to stand for 10 mins on ice. The supernatants were transferred to centrifuge tubes (Orange Scientific, Braine-l'Alleud | Belgium) and centrifuged at 10,000 g and 4°C for 15 min in an MR 23i centrifuge (Jouan Industries S.A.S., France). The supernatant was removed just after centrifugation with a glass Luer-lock syringe and filtered through a 13-mm-diameter, 0.45-µm regenerated cellulose filter (TR-200435, OmniPeak, Teknokroma, Barcelona, Spain).

### Purification

The 1 ml (column dimensions 0.7×2.5 cm) p-ABA column (17-5143-01, GE Healthcare) was eluted and equilibrated with twelve column volumes of binding buffer. The filtered homogenate was loaded dropwise onto the column, which was left standing in a cold room at 4°C for 20 min, and then the same volume of supernatant was loaded onto the column. After an additional 20 min, the column was cleaned with 20 volumes of binding buffer. Finally, twelve column volumes of elution buffer were used to elute purified protein. The purified proteins were cleaned using a PD MidiTrap G-25 according to the manufacturer's instructions. The protein content was measured using the Bradford reagent.

A 0.5 M solution of CaCl_2_ in 0.02 M Tris-HCl was prepared to remove calcium-dependent proteins, since phosphate buffer will react with CaCl_2_ to form nearly insoluble Ca_3_(PO_4_)_2_. The 1 mL p-ABA column was washed dropwise with 20 volumes of CaCl_2_ buffer. The column was then reequilibrated with fifteen column volumes of binding buffer. The following steps were the same as those described above.

### Lyophylization

The purified protein was divided into 1- to 2-mL aliquots, added to 15-mL centrifuge tubes, covered with a 0.22-µm PTFE filter (TR-200210, OmniPeak, Teknokroma) and fixed using a cap with a vent hole. The tubes were frozen and lyophilized in a PowerDry LL3000 lyophilizer (Thermo, Shanghai, China) and stored in the freezer at −40°C for later use.

### Separation of proteins using SDS-PAGE tris-glycine electrophoresis

The protein samples were separated using a SDS-PAGE tris-glycine electrophoresis system according to the manufacturer's instructions (Sigma-Aldrich). For electrophoresis, the proteins were diluted in two different sample buffers (with and without the reducing agent DTT), either with or without boiling step, respectively. The electrophoresis was performed under constant voltage in an SE 600 Ruby electrophoresis instrument (GE Healthcare) or MiniPROTEAN® Tetra Cell (Bio-Rad). The gel was fixed in fixing solution (40% LC-MS methanol, 10% ice acetic acid, 50% nanopure water) for two hours and stained with 0.02% PhastGel™ Blue R (GE Healthcare). Fixing solution was used to destain the gels.

### Separation of proteins using two-dimensional gel electrophoresis

Isoelectrofocusing (IEF) was performed on an Ettan IPG Phor 3 instrument (GE Healthcare). The separation was performed in 13 cm ceramic strip holders, using immobiline dry strips with a range of pH 3–10. A DeStreak Rehydration solution containing 0.5% IPG buffer pH 3–10 was used for active rehydration. The separation program was: 1) Step, 30 V, 10H; 2) Step 500 V, 500 Vh; 3) Grad 1000 V, 800 Vh; 4) Grad 6000 V, 15000 Vh; 5) Step 6000 V, 16000 Vh. The isoelectrofocusing program together with active rehydration ran for 19 h. Immediately following IEF, the strips were equilibrated for 15 min in equilibration buffer containing DTT and then for 15 min in buffer containing IAA. The strips were placed on a gel and fixed with 1% agarose. The electrophoresis was run at a constant voltage of 30 V for 50 min, after which the proteins were separated at a constant voltage of 300 V under cooling.

### Protein identification

The protein bands or spots were cut from the gel and analyzed by Proteome Factory AG, Berlin using nano LC-ESI-MS/MS. MASCOT software (Matrix Science, Boston, MA) was used to identify the protein MS/MS data, and the data were further compared against the NCBInr protein database. The following search parameters were used: trypsin was used as the digestion enzyme, the monoisotropic peptide window was set to unrestricted, zero missed cleavage was allowed, and propionamide was allowed as a variable modification. The peptide mass tolerance was 0.1%, the fragment mass tolerance was 0.6 Da, and the significance threshold was p<0.05.

Samples from the last purification step, wherein Ca^2+^ binding proteins were purified from *O. cuniculus* hind muscle were analyzed by the Laboratory of Mass Spectrometry, Faculty of Science, Charles University, Prague. The analyses were executed on a MALDI TOF/TOF mass spectrometer (Applied Biosystems), and the data were further searched against the NCBInr protein database, as were the ESI-Trap analyses.

## Results

### Extraction of proteins and purification

The purification method enabled a relatively high yield of purified protein from 2 mL of extracts. The protein yield from mites was approximately 3 mg of total protein as measured by Bradford, while the protein yield from *Blatella germanica* was approximately 5 mg. Approximately 4.5 mg of protein was obtained from *Oryctogalus cuniculus* muscle.

### Purification of tropomyosin, paramyosin and actins from mites

**Figure 1:**
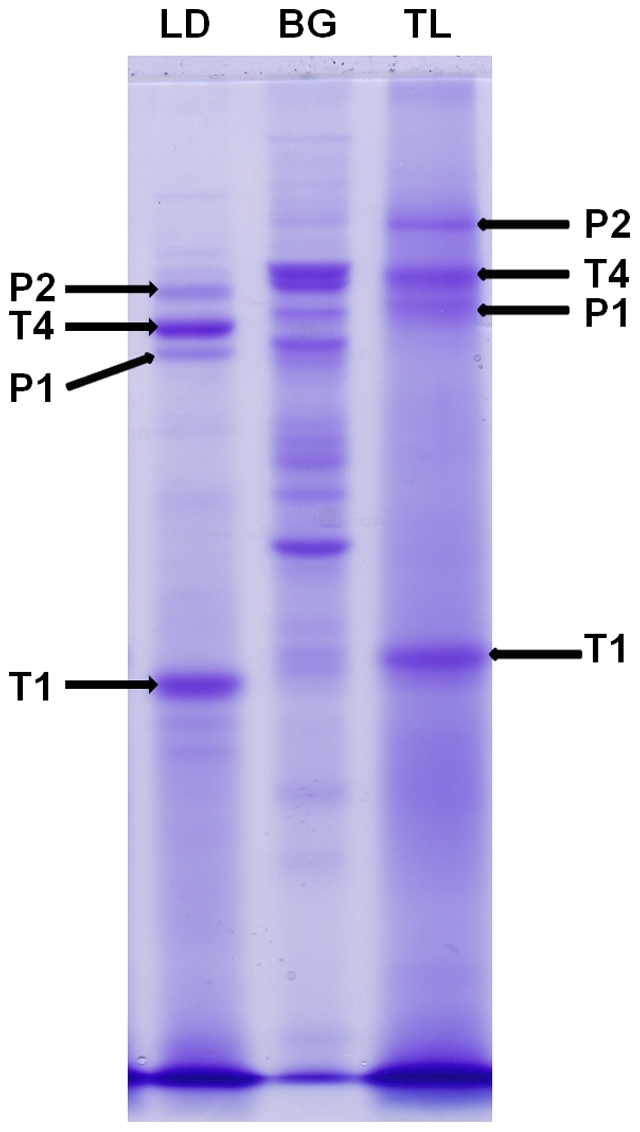
Non-reducing coomassie stained SDS-PAGE of proteins purified from *Lepidoglyphus destructor* (LD) and *Tyroborus lini* (TL), compared with proteins purified from *Blatella germanica* (BG), 60 µg protein per lane. Legend: T1 – tropomyosin monomer; T4 – tropomyosin tetramer; P1 – paramyosin monomer; P2 – paramyosin dimer.

**Figure 2:**
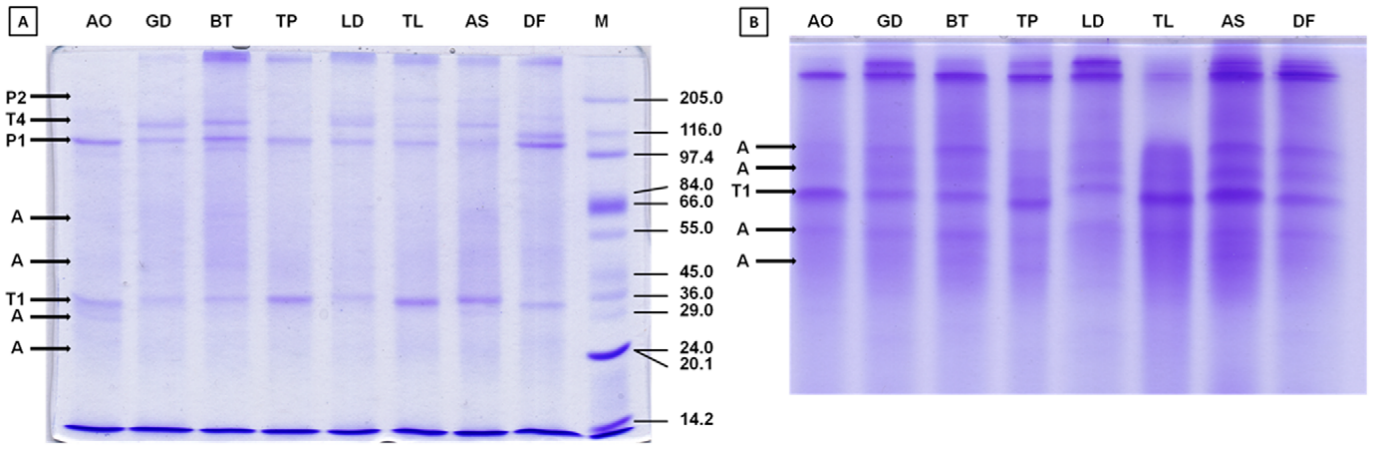
Coomassie stained tris-glycine 8×10 cm SDS-PAGE of proteins purified from eight species of synanthropic acaridid mites. A) Reducing 10% SDS-PAGE, 10 µg protein per lane; B) Reducing 20% SDS-PAGE, 10 µg protein per lane (figure has been shortened). Legend: A – actin; T1 – tropomyosin monomer; T4 – tropomyosin tetramer; P1 – paramyosin monomer; P2 – paramyosin dimer; AO – *Aleuroglyphus ovatus*; GD – *Glycyphagus domesticus*; BT – *Blomia tropicalis*; TP – *Tyrophagus putrescentiae*; LD – *Lepidoglyphus destructor*; TL – *Tyroborus lini*; AS – *Acarus siro*; DF – *Dermatophagoides farinae*; M – marker.

The method purified mostly tropomyosin and paramyosin from the crude mite extracts. The two protein bands of *L. destructor* were identified as allergen Lep d10 (mass 32930 Da; NCBI gi|14423956), while another two bands were identified as allergen Der f11 (mass 102325 Da; NCBI gi|42559514). Similarly, two proteins of *T. lini* were allergen Der f10 (mass 34671; NCBI gi|1359436) and allergen Der p11 (mass 98 kDa; NCBI gi|37778944), respectively. Thus, the results indicate that mite tropomyosin is monomeric or dimeric. In addition, the similar staining intensity of bands for monomer and tetramer on non-reducing SDS-PAGE (Figure 1) indicates that monomeric tropomyosin was separated in a four-fold less molar concentration as compared to the tetrameric form. Similarly, the results indicate monomeric and dimeric forms for paramyosin, with an analogous two-fold amount of the monomeric form compared to the dimeric form after non-reducing SDS-PAGE (Figure 1). The SDS-PAGE (Figure 2A) confirmed usability of the method for the purification of tropomyosin and paramyosin in eight species of acaridid mites. Since some additional bands were also visible near the band corresponding to monomeric tropomyosin (Figure 2A), the proteins were further separated using 20% SDS-PAGE (Figure 2B) to obtain sharper bands of smaller proteins. All four bands were identified as actin, with the highest similarity to actin from *Sarcoptes scabiei* type hominis (NCBI gi|186477888) (Figure 2AB).

To show the applicability to other invertebrate systems, one of the major producers of allergens in the human environment, *Blatella germanica*, was selected for comparison with the purified protein from mites. The protein profile (Figure 1, BG) purified from *B. germanica* was distinct from non-reducing SDS-PAGE obtained from mites. Therefore, the proteins from *B. germanica* were further analysed using two-dimensional approach.

### Analysis of proteins purified from *Blatella germanica*

**Figure 3:**
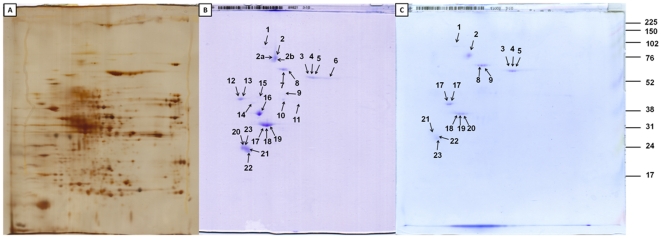
*Blatella germanica* two-dimensional gel electrophoresis. A) silver stained 16×18 cm electrophoreogram, proteins before purification, 200 µg of proteins. B) and C) Coomassie stained gels, proteins after purification. The two different repeats of purification are shown, 150 µg of proteins each. The numbers denote samples in table 1.

Two-dimensional gel electrophoresis was used to precisely identify proteins purified from *B. germanica* bodies separated from the abdomen. The two-dimensional gel electrophoresis results indicated that some proteins are probably present in more isoforms (Figure 3BC, Table 1). Tropomyosin and paramyosin together with actin were of primary interest because were the most abundant proteins identified in mite samples. The form of the paramyosin spot indicated that additional isoforms or perhaps other proteins could be located near paramyosin in two-dimensional gel electrophoresis. The analysis of different parts of the unsquared spot 2 (Table 1, sample 2, 2ab) confirmed paramyosin identification. Additional major purified proteins were actins and tubulins. Muscle myosin heavy chains and two isoforms of alpha amylase were identified as minor proteins. In conclusion, this experiment demonstrates that several types of proteins can be purified from *B. germanica* for proteomic studies. Comparison of the protein spectra before and after purification (Figures 3) demonstrates protein depletion for proteomic analysis of purified proteins.

**Table 1:** Proteins purified from *Blatella germanica* identified by nano LC ESI-MS/MS.

Spot	NCBI Blast	Taxonomy	Best Protein Description	Score	Mass
1	gi|183979376	*Papilio xuthus*	Muscle myosin heavy chain	774	224506
1	gi|156544337	*Nasonia vitripennis*	PREDICTED: similar to CG17927-PF	753	224506
2	gi|66510482	*Apis mellifera*	Similar to Paramyosin CG5939-PA, isoform A	306	101994
2a	gi|66510482	*Apis mellifera*	Similar to Paramyosin CG5939-PA, isoform A	485	101994
2a	gi|156542010	*Nasonia vitripennis*	PREDICTED: similar to standard paramyosin	409	102316
2b	gi|66510482	*Apis mellifera*	Similar to Paramyosin CG5939-PA, isoform A	288	101994
2b	gi|10959	*Drosophila melanogaster*	Paramyosin	200	102162
3	gi|28317	*Homo sapiens*	Unnamed protein product	50	59492
4	gi|193795848	*Bombyx mandarina*	Paramyosin	110	102668
5	gi|6981420	*Rattus norvegicus*	Anionic trypsin-1 precursor	64	25943
6	gi|195443610	*Drosophila willistoni*	GK11544	64	143510
7	gi|85002763	*Blattella germanica*	Alpha-amylase	205	56759
8	gi|66510482	*Apis mellifera*	Similar to Paramyosin CG5939-PA, isoform A	163	101994
9	gi|85002763	*Blattella germanica*	Alpha-amylase	619	56759
10	gi|187281831	*Bombyx mori*	Actin, muscle-type A2	85	41776
11	gi|195156301	*Drosophila persimilis*	GL26144	139	57322
12	gi|20069089	*Aplysia californica*	Alpha tubulin 2	162	50129
13	gi|85002763	*Blattella germanica*	Alpha-amylase	293	56759
14	gi|187281831	*Bombyx mori*	Actin, muscle-type A2	245	41776
14	gi|12585365	*Manduca sexta*	Tubulin beta-1 chain	674	50198
15	gi|21667231	*Ciona intestinalis*	Alpha-tubulin 3	536	53937
15	gi|32400724	*Oikopleura dioica*	Putative alpha-tubulin	536	50072
16	gi|187968785	*Goleba lyra*	Actin	513	22382
17	gi|187281831	*Bombyx mori*	Actin, muscle-type A2	676	41776
18	gi|113230	*Artemia sp.*	Actin, clone 211	548	41757
19	gi|187281831	*Bombyx mori*	Actin, muscle-type A2	735	41776
19	gi|155966246	*Lepeophtheirus salmonis*	Actin	766	41727
20	gi|4378573	*Periplaneta americana*	Tropomyosin	397	32775
21	gi|187281831	*Bombyx mori*	Actin, muscle-type A2	245	41776
22	gi|156773	*Drosophila melanogaster*	Actin	188	41748
23	gi|187281831	*Bombyx mori*	Actin, muscle-type A2	140	41776

### Analysis of proteins purified from *Oryctogalus cuniculus* hind muscle

**Figure 4:**
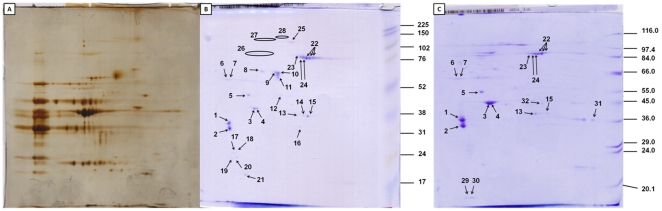
*Oryctogalus cuniculus* two-dimensional gel electrophoresis after purification. A) silver stained 16×18 cm SDS-PAGE before purification, 200 µg proteins; Coomassie stained gels, 150 µg proteins each; B) 10% and C) 12%, after purification. The numbers in gels denote sample in table 2.

The usefulness of the method was tested on a pure muscle vertebrate tissue sample using the same approach as described above. The experiment was executed on a cadaver sample that was currently available in the laboratory. Similarly to the analysis of the *B. germanica* sample, the major product was tropomyosin, while paramyosin, an exclusive invertebrate protein, was not detected (Table 2). The analysis confirmed the presence of the alpha and beta chains of tropomyosin, the most abundant proteins in two-dimensional gel electrophoresis, with similar intensity, indicating comparable amounts of both proteins. The major purified proteins from *O. cuniculus* hind muscle were tropomyosin alpha and beta chain, but two isoforms of skeletal muscle alpha actin 2 were also present in large amounts. Many highly abundant isoforms of glycogen phosporylase b have been also identified. Four additional isoforms were identified as 14-3-3 proteins (Table 2). Two representative 10% (Figure 4B) and 12% (Figure 4C) coomassie-stained gels of purified proteins are shown. The 12% gel allows to discriminate more proteins than the 10% gel, therefore several other proteins are visible, including two isoforms of a 19-kDa myosin regulatory light chain 2. The depletion of proteins by the purification process enables proteomic studies of the identified proteins. Most of the proteins identified were taxonomically assigned to *O. cuniculus*, indicating a high level of authenticity for the identification.

**Table 2:** Nano LC ESI-MS/MS-identified proteins after purification from *Oryctogalus cuniculus* hind muscle.

Spot	NCBI Blast	Taxonomy	Best Protein Description	Score	Mass
1	gi|11875203	*Mus musculus*	Tropomyosin beta chain	640	32817
2	gi|230767	*Oryctolagus cuniculus*	Chain A, Tropomyosin	643	32661
3	gi|157144153	*Pagrus major*	Skeletal muscle alpha actin 2	784	41978
4	gi|157144153	*Pagrus major*	Skeletal muscle alpha actin 2	739	41978
5	gi|194037554	*Sus scrofa*	PREDICTED: similar to ATP synthase subunit beta, mitochondrial	1541	56300
6	gi|2950347	*Mus musculus*	M-protein	334	164416
7	gi|20988232	*Mus musculus*	Myosin binding protein C-fast type	470	127245
8	gi|231257	*Oryctolagus cuniculus*	Chain A, Structural Mechanism For Glycogen Phosphorylase Control By Phosphorylation And Amp	646	97097
9	gi|126723746	*Oryctolagus cuniculus*	Serum albumin precursor	294	68865
10	gi|126723746	*Oryctolagus cuniculus*	Serum albumin precursor	390	68865
11	gi|223003	*Oryctolagus cuniculus*	Phosphorylase b,glycogen	691	96998
12	gi|623545	*Oryctolagus cuniculus*	Sarcoplasmic reticulum glycoprotein	608	54416
13	gi|73535342	*Oryctolagus cuniculus*	Chain A, Transition State Analog Complex Of Muscle Creatine Kinase (R134k) Mutant	614	42926
14	gi|73535342	*Oryctolagus cuniculus*	Chain A, Transition State Analog Complex Of Muscle Creatine Kinase (R134k) Mutant	591	42926
15	gi|73535342	*Oryctolagus cuniculus*	Chain A, Transition State Analog Complex Of Muscle Creatine Kinase (R134k) Mutant	688	42926
16	gi|73535342	*Oryctolagus cuniculus*	Chain A, Transition State Analog Complex Of Muscle Creatine Kinase (R134k) Mutant	288	42926
17	gi|3065929	*Mus musculus*	14-3-3 protein gamma	371	28345
18	gi|3065929	*Mus musculus*	14-3-3 protein gamma	407	28345
19	gi|530049	*Ovis aries*	14-3-3 protein	526	26279
20	gi|47086819	*Danio rerio*	14-3-3 protein epsilon	254	29054
21	gi|1096612	*Bos taurus*	Myosin:SUBUNIT = light chain 1	455	18671
22	gi|223003	*Oryctolagus cuniculus*	Phosphorylase b, glycogen	1403	96998
22	gi|223003	*Oryctolagus cuniculus*	Phosphorylase b, glycogen	1384	96998
22	gi|223003	*Oryctolagus cuniculus*	Phosphorylase b, glycogen	1114	96998
23	gi|223003	*Oryctolagus cuniculus*	Phosphorylase b, glycogen	893	96998
24	gi|231257	*Oryctolagus cuniculus*	Chain A, Structural Mechanism For Glycogen Phosphorylase Control By Phosphorylation And Amp	1521	97097
24	gi|231257	*Oryctolagus cuniculus*	Chain A, Structural Mechanism For Glycogen Phosphorylase Control By Phosphorylation And Amp	1161	97097
25	gi|20988232	*Mus musculus*	Myosin binding protein C, fast-type	603	127245
26	gi|231257	*Oryctolagus cuniculus*	Chain A, Structural Mechanism For Glycogen Phosphorylase Control By Phosphorylation And Amp	977	97097
27	gi|20988232	*Mus musculus*	Myosin binding protein C, fast-type	470	127245
28	gi|2950347	*Mus musculus*	M-protein	334	164416
29	gi|127176	*Oryctolagus cuniculus*	Myosin regulatory light chain 2, skeletal muscle isoform type2	198	19014
30	gi|127176	*Oryctolagus cuniculus*	Myosin regulatory light chain 2, skeletal muscle isoform type2	198	19014
31	gi|40889050	*Oryctolagus cuniculus*	Chain O, Crystal Structure Of The Rabbit Muscle Glyceraldehyde-3-Phosphate Dehydrogenase	311	35678
32	gi|65987	*Sus scrofa domestica*	Glyceraldehyde-3-phosphate dehydrogenase (phosphorylating) (EC 1.2.1.12)	253	35686

### Analysis of proteins purified from *Oryctogalus cuniculus* hind muscle after the removal of calcium-dependent proteins

**Figure 5:**
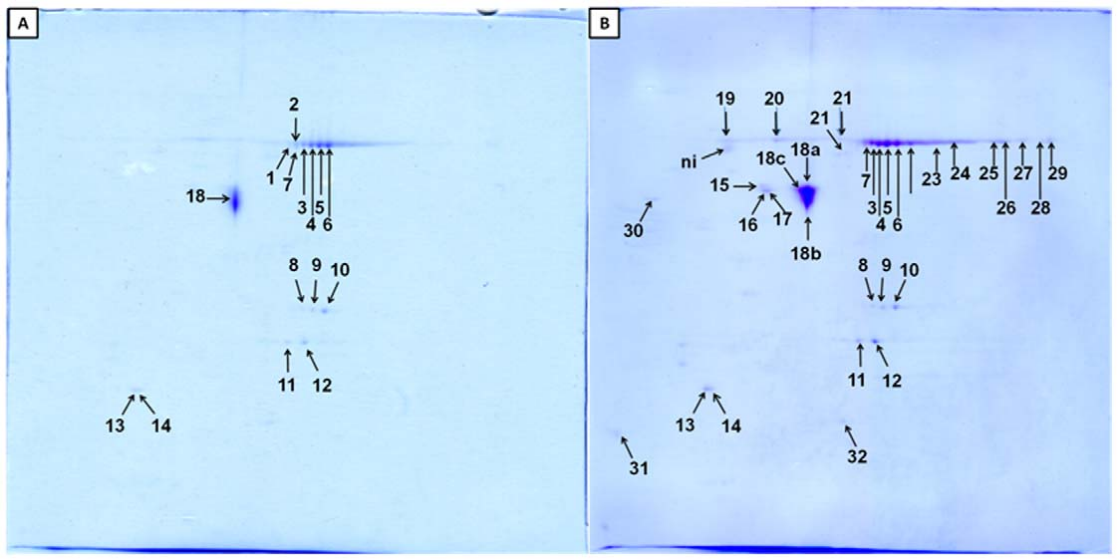
*Oryctogalus cuniculus* two-dimensional coomassie stained gel electrophoresis after removal of calcium-dependent proteins, A) 50 µg protein; B) 75 µg protein with less destaining. The numbers in gels denote sample in table 3.

A 1 M CaCl_2_ solution in 0.1 M Tris-HCl buffer, adjusted to pH 7.4 with HCl, was used to remove calcium-dependent proteins. The following two-dimensional analyses showed that tropomyosin, actin and tubulin were removed following this step. The most interesting proteins further identified were kinases and annexins that were present in more isoforms (Figure 5). The complete list of identified proteins is included as table 3.

**Table 3:** MALDI TOF/TOF-identified proteins after depletion of calcium-dependent protein from *Oryctogalus cuniculus* hind muscle.

Spot	NCBI Blast	Taxonomy	Best Protein Description	Score	Mass
1	gi|66361339	*Oryctolagus cuniculus*	Chain A, Glycogen Phosphorylase Amp Site Inhibitor Complex	434	97593
2	gi|1827888	*Oryctolagus cuniculus*	Chain A, Complex Of Glycogen Phosphorylase With A Transition State Analogue Nojirimycin Tetrazole And Phosphate In The T And R States	375	97636
3	gi|66361339	*Oryctolagus cuniculus*	Chain A, Glycogen Phosphorylase Amp Site Inhibitor Complex	671	97593
4	gi|6730143	*Oryctolagus cuniculus*	Chain A, Identification And Structural Characterization Of A Novel Allosteric Binding Site Of Glycogen Phosphorylase B	748	96157
5	gi|66361339	*Oryctolagus cuniculus*	Chain A, Glycogen Phosphorylase Amp Site Inhibitor Complex	772	97593
6	gi|6730143	*Oryctolagus cuniculus*	Chain A, Identification And Structural Characterization Of A Novel Allosteric Binding Site Of Glycogen Phosphorylase B	863	96157
7	gi|1827888	*Oryctolagus cuniculus*	Chain A, Complex Of Glycogen Phosphorylase With A Transition State Analogue Nojirimycin Tetrazole And Phosphate In The T And R States	664	97636
8	gi|126723370	*Oryctolagus cuniculus*	Creatine kinase M-type	422	43313
9	gi|126723370	*Oryctolagus cuniculus*	Creatine kinase M-type	515	43313
10	gi|126723370	*Oryctolagus cuniculus*	Creatine kinase M-type	783	43313
11	gi|259912	*Oryctolagus cuniculus*	Troponin T beta isoform, TnT beta isoform, TnT-5	76	29665
12	gi|259912	*Oryctolagus cuniculus*	Troponin T beta isoform, TnT beta isoform, TnT-5	298	29665
13	gi|291401824	*Oryctolagus cuniculus*	PREDICTED: annexin 5	535	37313
14	gi|291401824	*Oryctolagus cuniculus*	PREDICTED: annexin 5	588	37313
15	gi|225632	*Bos taurus*	Casein alphaS1	144	24477
16	gi|291387656	*Oryctolagus cuniculus*	PREDICTED: annexin VI isoform 1	451	76102
17	gi|291387656	*Oryctolagus cuniculus*	PREDICTED: annexin VI isoform 1	614	76102
18a	gi|126723746	*Oryctolagus cuniculus*	Serum albumin precursor	848	70861
18b	gi|126723746	*Oryctolagus cuniculus*	Serum albumin precursor	603	70861
18c	gi|126723746	*Oryctolagus cuniculus*	Serum albumin precursor	716	70861
19	gi|1827888	*Oryctolagus cuniculus*	Chain A, Complex Of Glycogen Phosphorylase With A Transition State Analogue Nojirimycin Tetrazole And Phosphate In The T And R States	382	97636
20	gi|1827888	*Oryctolagus cuniculus*	Chain A, Complex Of Glycogen Phosphorylase With A Transition State Analogue Nojirimycin Tetrazole And Phosphate In The T And R States	855	97636
21	gi|1827888	*Oryctolagus cuniculus*	Chain A, Complex Of Glycogen Phosphorylase With A Transition State Analogue Nojirimycin Tetrazole And Phosphate In The T And R States	855	97636
22	gi|1827888	*Oryctolagus cuniculus*	Chain A, Complex Of Glycogen Phosphorylase With A Transition State Analogue Nojirimycin Tetrazole And Phosphate In The T And R States	338	97636
23	gi|1827888	*Oryctolagus cuniculus*	Chain A, Complex Of Glycogen Phosphorylase With A Transition State Analogue Nojirimycin Tetrazole And Phosphate In The T And R States	742	97636
24	gi|1827888	*Oryctolagus cuniculus*	Chain A, Complex Of Glycogen Phosphorylase With A Transition State Analogue Nojirimycin Tetrazole And Phosphate In The T And R States	705	97636
25	gi|291392785	*Oryctolagus cuniculus*	PREDICTED: enolase 2-like	353	47536
26	gi|1827888	*Oryctolagus cuniculus*	Chain A, Complex Of Glycogen Phosphorylase With A Transition State Analogue Nojirimycin Tetrazole And Phosphate In The T And R States	746	97636
27	gi|1827888	*Oryctolagus cuniculus*	Chain A, Complex Of Glycogen Phosphorylase With A Transition State Analogue Nojirimycin Tetrazole And Phosphate In The T And R States	388	97636
28	gi|1827888	*Oryctolagus cuniculus*	Chain A, Complex Of Glycogen Phosphorylase With A Transition State Analogue Nojirimycin Tetrazole And Phosphate In The T And R States	582	97636
29	gi|6730143	*Oryctolagus cuniculus*	Chain A, Identification And Structural Characterization Of A Novel Allosteric Binding Site Of Glycogen Phosphorylase B	355	96157
30	gi|126723562	*Oryctolagus cuniculus*	Calreticulin precursor	372	48416
31	gi|30722443	*Mus musculus*	SH3 domain-binding glutamic acid-rich protein	308	14581
32	gi|1364243	*Oryctolagus cuniculus*	Unnamed protein product	259	30973

## Discussion

A p-Aminobenzamidine (p-ABA) ligand enabled the purification of non-protease proteins, with variable specificity depending on the tissue source and slight modifications to the purification process. Although the p-ABA column is used to purify proteases, no proteases were purified from any of the analyzed samples. Moreover, because p-ABA binds to calcium-dependent proteins, and those with binding sites containing serine, it is not a suitable inhibitor of proteolysis during the purification of such proteins. Panallergenic tropomyosin and paramyosin were purified from eight species of house dust and stored product mites. This enables the testing of the native forms of these proteins for allergenicity and obviates the need for recombinant mite tropomyosin or tropomyosin in these tests and in vaccines. It was shown, for the first time, that mite tropomyosin is active in monomeric and tetrameric forms, while paramyosin is a monomeric and dimeric protein. The specificity of the method for tropomyosin and paramyosin was confirmed for *Blatella germanica*. The method was applied to rabbit hind muscle to determine its specificity in pure tissue. The purified proteins were, i.e. tropomyosins, actins, troponins, tubulins, creatine kinases, 14-3-3 proteins, glycogen phosphorylases b and annexins. The removal of calcium-dependent proteins from rabbit hind muscle pointed out even more selective purification. It is possible to obtain two purified samples: a) calcium dependent proteins and b) calcium independent proteins. The great advantage of the entire method, which may produce native protein for study in a variety of fields, is nondestructive. The method is applicable to various scientific branches, but is especially practical for medicinal applications. Moreover, p-ABA may be useful as a model to study processes involving the phosphorylation of serine.

It was demonstrated that a p-ABA column can isolate tropomyosin and paramyosin from eight species of mites, with high affinity also observed for mite actin, which can be separated using 20% SDS-PAGE. Further analysis on the *B. germanica* and *O. cuniculus* samples confirmed the high specificity of the p-ABA ligand for these proteins. The function and allergenicity of both of these proteins have been extensively studied. Tropomyosin and paramyosin are found in the muscles of a wide variety of animals, but paramyosin is found exclusively in invertebrates [9], [10], [11]. Tropomyosin is a two-chain α-helical coiled coil whose partially periodic interactions with the F-actin helix for thin filament stabilization and the regulation of muscle contraction [12]. Similarly to tropomyosin, paramyosin is α-helical coiled coil protein. Paramyosin is known as an antigen during infections by several flatworms that are parasites of humans and animals [13], [14]. In general, invertebrate tropomyosins and paramyosins represent major allergen groups (see the IUIS list of allergens; http://www.allergen.org) and are considered panallergens since they are highly conserved and cross-reactive in many invertebrates [10], [11], [15], [16], [17].

After lyophylization, the purified, desalted mite proteins are ready for use in allergenicity screens, and might also be suitable for use in vaccines or production of antibodies. Previously used purification methods include anionic exchange chromatography, gel chromatography and antibody columns. In some assays, boiling steps and p-ABA was used to prevent proteolysis [18], [19], [20], [21]. Here, it is suggested that p-ABA is not a convenient inhibitory agent in purification processes, since it binds these proteins. These proteins are usually produced by expression systems for research use, but native and recombinant proteins do not always show the same IgE reactivity. For example, the IgE reactivities of recombinant full-length Blo t11 allergen and its native form differ [18]. Here, it is suggested that the allergens purified from the original source could be more useful than recombinant allergens, which still require purification steps, and do not include native posttranslational modifications from original source.

The results also show acaridid mite tropomyosin and paramyosin oligomeric forms that were unknown until now [11]. Tropomyosin is present in monomeric and terameric forms, while paramyosin is monomeric and dimeric. These features are important, since the allergenicity of monomeric and n-meric structures may differ. Two key factors in the varying allergenicity of n-meric structures are different molar concentrations of the allergen, and the fact that n-meric structures can have hidden or activated epitopes compared to the monomer. For instance, if the allergen is present in tetramer, the concentration is four-fold less as compared to the monomeric form.

The two-dimensional gel electrophoresis of *B. germanica* proteins showed that the method purified tropomyosin and paramyosin as well as other muscle proteins, mainly actin and tubulin. It is interesting that alpha amylase was identified in the electrophoreogram of the *B. germanica* sample because it suggests that these enzymes can be purified from the *B. germanica* gut. The source of alpha amylase was probably very limited, as the isoenzymes are localized in the chest or salivary glands. This insect provides a challenge for future studies and further application of the method.

Finally, proteomic analysis of proteins purified from the *O. cuniculus* hind muscle confirmed the high affinity of p-ABA for tropomyosin (paramyosin is not produced by vertebrates). Two major spots, the alpha and beta chains of tropomyosin, were easily recognized in similar abundance after the depletion of proteins that were not of interest. Similarly, skeletal muscle alpha actin 2, glycogen phosporylase b and 14-3-3 proteins can be studied after the depletion process. The four isoforms of 14-3-3 proteins that were identified after depletion are of interest to the study of human disease, particularly cancer research [22]. All 14-3-3 proteins bind to common phosphoserine/phosphothreonine-containing peptide motifs, corresponding to Mode-1 (RS*X*pS*X*P) or Mode-2 (R*XXX*pS*X*P) sequences, where ‘X’ denotes any amino acid residue and pS denotes phosphorylated serine [23]. I suggest that p-ABA simulates phosphorylated serine, allowing it to bind 14-3-3 proteins and other phoshorylated proteins.

In the last step, Ca^2+^ wash buffer was used to remove calcium-binding proteins. This last step has two practical benefits: calcium-binding proteins were separated out as a group, and additional proteins, that is, creatine kinases and annexins, were then visible in electrophoretogram. The removed calcium-dependent proteins could be studied using a chemiproteomics approach also.

The most abundant proteins purified in the last step from *O. cuniculus* hind muscle were isoforms of glycogen phosphorylase b and other associated proteins. Glycogen phosporylase b is a substrate for phosphorylase b kinase, which catalyzes the phosphorylation of serine in certain substrates including troponin I. The beta chain acts as a regulatory unit and modulates the activity of the holoenzyme in response to phosphorylation [24]. Here, as in the case of 14-3-3 proteins, p-ABA probably simulated phosphorylated serine. Creatine kinase type-m was easily recognized after the removal of calcium-dependent proteins. This kinase reversibly catalyzes the transfer of phosphate between ATP and various phosphogens (e.g., creatine phosphate). Creatine kinase isoenzymes play a central role in energy transduction in tissues with large, fluctuating energy demands, such as skeletal muscle, heart, brain and spermatozoa [25].
